# Trade‐offs for butterfly alpha and beta diversity in human‐modified landscapes and tropical rainforests

**DOI:** 10.1002/ece3.4732

**Published:** 2018-12-06

**Authors:** Hemchandranauth Sambhu, Alliea Nankishore, Stephen M. Turton, Tobin D. Northfield

**Affiliations:** ^1^ College of Science and Engineering James Cook University Smithfield Queensland Australia; ^2^ Department of Biology, Faculty of Natural Sciences University of Guyana, Turkeyen Greater Georgetown Guyana; ^3^ Georgetown Guyana; ^4^ Central Queensland University Cairns City Queensland Australia; ^5^ Department of Entomology, Tree Fruit Research and Extension Center Washington State University Wenatchee Washington

**Keywords:** butterfly conservation, land management practices, landscape approaches to biodiversity conservation, sugarcane cultivation, urban green spaces, Wet Tropics

## Abstract

The accelerating expansion of human populations and associated economic activity across the globe have made maintaining large, intact natural areas increasingly challenging. The difficulty of preserving large intact landscapes in the presence of growing human populations has led to a growing emphasis on landscape approaches to biodiversity conservation with a complementary strategy focused on improving conservation in human‐modified landscapes. This, in turn, is leading to intense debate about the effectiveness of biodiversity conservation in human‐modified landscapes and approaches to better support biodiversity in those landscapes. Here, we compared butterfly abundance, alpha richness, and beta diversity in human‐modified landscapes (urban, sugarcane) and natural, forested areas to assess the conservation value of human‐modified landscapes within the Wet Tropics bioregion of Australia. We used fruit‐baited traps to sample butterflies and analyzed abundance and species richness in respective land uses over a one‐year period. We also evaluated turnover and spatial variance components of beta diversity to determine the extent of change in temporal and spatial variation in community composition. Forests supported the largest numbers of butterflies, but were lowest in each, alpha species richness, beta turnover, and the spatial beta diversity. Sugarcane supported higher species richness, demonstrating the potential for conservation at local scales in human‐modified landscapes. In contrast, beta diversity was highest in urban areas, likely driven by spatial and temporal variation in plant composition within the urban landscapes. Thus, while improving conservation on human‐modified landscapes may improve local alpha richness, conserving variation in natural vegetation is critical for maintaining high beta diversity.

## 1 INTRODUCTION

In response to a growing and expanding human population, natural habitats and the landscape as a whole are increasingly being shaped by human activities (Venter et al., 2016). McGill, Dornelas, Gotelli, and Magurran (2015) identified five major ways that human activities impact biodiversity: land‐cover change, chemical release, overharvesting, climate change, and species transport/invasion. These transformative activities are multi‐dimensional and are often conducted for economic and social gains. One of the main drivers of land‐cover change is the clearing of lands for agriculture and urbanization (DeFries, Rudel, Uriarte, & Hansen, 2010; Kissinger, Herold, & DeSy, 2012), and these are projected to continue expanding in the coming years (Schmitz et al., 2014; Seto, Fragkias, Güneralp, & Reilly, 2011). Up to 92% of densely forested areas are reportedly suitable for agriculture (Zabel, Putzenlechner, & Mauser, 2014), and urbanization is projected to increase in all habitat types (McDonald, Marcotullio, & Güneralp, 2013; Seto, Güneralp, & Hutyra, 2012). There will therefore be continued pressure on natural spaces to give way for food production and housing.

It is evident that these activities result in declines in biodiversity. Johnson et al. (2017) highlight some of the trends in extinction across different animal groups and landscapes. They note that this loss in biodiversity affects the functioning of natural ecosystems and the environmental services they provide and, in so doing, also threatens human wellbeing. So, the quest to increase agricultural land and urban living space may also be seen as a Catch‐22, as there is a wealth of knowledge that suggests that human wellbeing depends on functioning environmental services and is closely linked to access to nature (biodiversity and green spaces) (Kilpatrick, Salkeld, Titcomb, & Hahn, 2017; Maller, Townsend, Pryor, Brown, & St. Leger, L., 2005; Sandifer, Sutton‐Grier, & Ward, 2015). However, that same activity (human development) has the potential to degrade the same natural ecosystem.

The primary method to safeguard nature by governments, non‐governmental organizations (NGOs), and individuals has been to increase the number and size of conservation areas and green spaces in human‐modified landscapes. However, these strategies are not without their problems, as there are issues of financing the operations involved and securing conservation land spaces in the ever‐expanding world of urbanization and agriculture. There is also the ever‐present question of the effectiveness of these strategies for conservation relative to improving conservation within human‐modified areas (Gray et al., 2016; Watson, Dudley, Segan, & Hockings, 2014).

Conversion to agriculture and urbanization can greatly reduce local species richness and abundance, although the impacts also depend on the intensification of these factors (Newbold et al., 2015). In addition to local changes in biodiversity, intensified land use can degrade beta diversity, particularly over large scales (Flohre et al., 2011; Gossner et al., 2016; Karp et al., 2012). For example, Karp et al. (2012) found that bird beta diversity measured as the turnover in bird communities over large spaces was lower in intensified agriculture than forest or low‐intensity agriculture. Similarly, Flohre et al. (2011) found that agricultural intensification reduced beta diversity (measured as spatial variation in community composition) at the farm and region scale of plants, birds, and carabid beetles, while effects on local diversity were often insignificant. Thus, the effects of land‐use change on beta diversity may be even greater at the local scale due to landscape homogeneity.

Generally, governments, NGOs, and individuals agree that there is still a great need for conservation spaces in this era (Ekkel & de Vries, 2017; Gill, Handley, Ennos, & Pauleit, 2007; Virtudes, 2016). Several have been created across the globe—one such space is the Wet Tropics bioregion in Australia, which extends over 500 km south along Queensland's north‐eastern coast from Cooktown to Townsville, and up to 50 km inland (Bohnet & Smith, 2007). Occupying less than 1% of the State of Queensland, this bioregion has the highest level of biodiversity in Australia and is an internationally recognized biodiversity hotspot (Stork, Goosem, & Turton, 2011), with approximately 48% of its rainforests having World Heritage status since 1988 (Bohnet & Smith, 2007; Stork, Goosem, & Turton, 2008).

The Wet Tropics bioregion is a multiple use area, with urban settlements and agricultural lands interspersed among strictly protected forested areas. Sugarcane (*Saccharum officinarum* L., 1753) is the major agricultural crop in the Wet Tropics (Kroon, Thorburn, Schaffelke, & Whitten, 2016), with its cultivation perceived by many as a threat to surrounding ecosystems. Several studies (Armour, Hateley, & Pitt, & G. L., 2009; Brodie & Mitchell, 2005; Haynes, Müller, & Carter, 2000a; Haynes, Ralph, Prange, & Dennison, 2000b; Kroon et al., 2016; Lewis et al., 2009; Mitchell, Brodie, & White, 2005; Tsatsaros, Brodie, Bohnet, & Valentine, 2013) have shown either direct impacts or threats of pesticide, nutrient and sediment runoff from sugarcane cultivation on different components of nearby coastal and marine systems. Additionally, monoculture plantations are known to be highly dissimilar from natural habitats in both composition and structure (Anand, Krishnaswamy, Kumar, & Bali, 2010). However, field margins within this landscape may help to support butterflies (Sambhu, Northfield, Nankishore, Ansari, & Turton, 2017), as has been found in other agricultural landscapes (Fahrig et al., 2015; Feber, Smith, & Macdonald, 1996; Hodgson, Kunin, Thomas, Benton, & Gabriel, 2010; Sybertz, Matthies, Schaarschmidt, Reich, & Haaren, 2017). We were interested in the conservation implications of the Wet Tropics management system, which allows for both ecosystem protection and landscape modification for livelihood and/or economic gains.

Given that it is difficult to study all biodiversity, indicator groups or species are routinely used to gain an understanding of the status of the environment. Butterflies are a suitable and popular group for biodiversity studies as their relatively well‐known taxonomy, geographic distribution, status, and sensitivity to environmental conditions make them ideal biological indicators (Blair, 1999; Padhye, Shelke, & Dahanukar, 2012). Butterfly diversity often decreases with greater urbanization (e.g., Blair, 1999) and agricultural intensification (e.g., Rundlöf & Smith, 2006; Hodgson et al., 2010), but can benefit from weedy margins within agricultural landscapes (Hodgson et al., 2010; Koh, 2008). Here, we investigated butterfly abundance, richness, evenness, and diversity in the Wet Tropics bioregion, in three different land uses: one natural (forested) areas and two human‐modified (urban and agricultural) areas. We expected that forests would serve as the best environment for butterfly populations. However, given the ability of farm margins to support butterfly populations in tropical habitats (e.g., Koh, 2008), we also hypothesized that agricultural areas may host a diverse group of butterflies. We expected these populations to be lower in beta diversity than forests and urban areas, given the low variation in plant composition in sugarcane farms, including the weedy field margins that support butterflies. Given that sugarcane is generally irrigated year‐round and mowed regularly in the study region, we also expected to find little temporal variation in species richness and abundance or little turnover in that landscape.

## 2 METHODS

### 2.1 Study area

Our study was conducted in the coastal lowlands of the northern half of the Wet Tropics bioregion of Far North Queensland, from Daintree in the north to Wooroonooran in the south (Supporting Information Appendix S1). The vegetation types consist of predominantly rainforest, along with sclerophyll forests and woodlands, sclerophyll and sclerophyll rainforest transitions, mangrove forests, shrubs and heathlands, vegetation complex and mosaics, non‐woody vegetation, and unvegetated/cleared land (WTMA, 2012a). The urban landscapes of the bioregion are a mosaic of low‐, medium‐ and high‐density settlements with a high degree of tree cover in close proximity to extensive natural forested areas (Turton, 2016). European settlement began in the 1870s, notwithstanding 50,000 years of Indigenous habitation of the bioregion (Turton, 2008). Many industries were established in the study area, all with differing consequences for the environment. These included the mining and dairy industries, sugarcane farming, and other tropical crops. Thus, land‐use types in the region generally include conservation, forestry, grazing, dairy, horticulture, cropping, and urban (Terrain, 2016).

The climate consists of one wet season between November and March (temperature 30–35°C; average rainfall 1,800–2,400 mm), and one dry season between April and October (temperature 17–29°C; average rainfall 600–1,200 mm) (Australian Government, 2015; Bureau of Meteorology, 2017). However, heavy rain can occur even during the dry season due to orographic uplift of prevailing southeast trade winds during that time of year.

Study sites were selected in areas with both natural (forested area >10 km^2^) and human‐modified landscapes (cropping, specifically sugarcane monocrop plantations, >10 km^2^ and urban settlements with human population >1,000 persons per 10 km^2^). Sugarcane farming has persisted in the area since the late 1800s (Griggs, 2000), and the farms we sampled from were well‐established farms that had been farmed for multiple decades. They were grouped broadly as (1) Gordonvale, (2) Smithfield, and (3) Mossman, with the land uses located as follows:

1. Wooroonoran National Park (forest), Gordonvale (sugarcane), Edmonton and Bentley Park (urban);
2. Smithfield Conservation Park (forest), Freshwater and Redlynch (sugarcane), Kewarra Beach, Trinity Beach and Redlynch (urban);
3. Daintree National Park (forest), Lower Daintree (sugarcane), Mossman and Port Douglas (urban).

Areas sampled included mainly mesophyll rainforests in the Daintree National Park, a mixture of notophyll rainforests and eucalyptus forests in the Smithfield Conservation Park, and notophyll rainforests in the Wooroonooran National Park (WTMA, 2012b). Of these forest types, mesophyll rainforests are the most developed or oldest (WTMA, 2012c). Canopy height for all of the surveyed forests is above 20 m, with canopy coverage greater than 70%.

In forested areas, we worked with local rangers to avoid areas of Indigenous cultural significance, or high traffic (e.g., mountain bike trails), and nonrandomly selected the remaining trails to place the transects. To select the locations of sugarcane transects, we worked with sugarcane growers to place transects nonrandomly along field margins. Urban transects were selected nonrandomly within the identified region, in accordance with permission from land‐owners.

### 2.2 Sampling of butterflies

Three 1 km transects were placed 1–1.5 km apart in each of the land‐use zones and beginning at least 100 m from the hard edge of each zone (Supporting Information Appendix S1). Those in the forests were laid out along existing trails (and followed straight lines when possible) so as to minimize disturbances to butterfly behavior and other forest users. Those in sugarcane plantations were established along headlands/field margins in an effort to reduce the impact of the research on the farmers’ crop and activities (e.g., cultivation and harvesting), while those in urban areas were established in green open spaces or in grassy areas surrounding homes. Each transect was visited monthly for 12 months (starting from June, 2016 and ending in May, 2017).

A total of 11 butterfly traps were placed 100 m apart in each transect, starting at the 0 m marker and ending at the 1 km marker, and each was labeled with a unique number and geo‐referenced. The traps were placed approximately 1.5 m above ground to ensure easy access and baited with approximately 100 g of a fermented mixture of bananas (*Musa *sp. L., 1753), 4.7 percent alcohol per volume of 275 ml beer and brown sugarcane sugar (4.5 kg of banana +4 beers +1 kg of sugar) (Nyafwono, Valtonen, Nyeko, & Roininen, 2014; Sambhu, 2009; Sambhu et al., 2017). They were checked daily between 08:00 hr and 16:00 hr over a three‐day period every month to reduce the bias of daily temperature fluctuation (Sands & New, 2002). Traps were re‐baited on an as‐needed basis during the three‐day checking period.

The stratification and ecological niches of various butterfly species make it difficult to capture all species present. However, fruit‐baited traps are one of the most reliable and unbiased methods for sampling tropical fruit‐feeding butterflies (Daily & Ehrlich, 1995; Hughes, Daily, & Ehrlich, 1998). Sampling at this level allowed for comparisons (Francesconi, Nair, Levey, Daniels, & Cullen, 2013) among the three contrasting land uses under investigation. Canopy butterfly species are often distinct from ground‐level species (Aduse‐Poku et al., 2012; Dumbrell & Hill, 2005) and were unlikely to be collected in our traps, so the issue of stratification (forests with tree canopy, sugarcane plantations with no canopy and urban sites with varying presence/level of canopy) was reduced. However, some primarily canopy‐dwelling butterflies are not exclusive to canopies (Aduse‐Poku et al., 2012) and are attracted to ground‐level fruit baits, so this trapping method also does not completely exclude canopy‐dwelling butterflies.

A catch‐and‐release method was used to sample butterfly diversity, with identifications done at the trap sites. When this was not possible, photographs were taken to assist with identification at a later time. Butterflies were identified with the aid of field guides covering the study region (Braby, 2004, 2016).

### 2.3 Data analyses

Migratory species, singletons and doubletons, were included in our analyses to account for the possibility of unknown factors affecting the presence of some butterflies during the sampling period (DeVries & Walla, 2001), as well as any methodological limitations that inadvertently exclude individuals, genuinely small populations and/or low individual numbers across narrow scales (Novotný & Basset, 2000).

To evaluate patterns in abundance and location, across the different land‐use types and locations, we used generalized linear mixed models with fixed effects of land use, location, and an interaction between land use and location. These analyses were undertaken using the “lme4” package in R v. 3.4 (R Core Development Team, 2018). Traps within transects were combined within a transect for analysis, such that transect was the experimental unit. We also included a random effect of month, and a random effect of transect to account for the fact that each transect was resampled multiple times. Preliminary analyses suggested there was no difference between wet and dry seasons in either metric, so this was not included. For the model describing abundance, we log_10_(*x* + 1) transformed the data and assumed a Gaussian distribution. This is due to over dispersion relative to a Poisson distribution, and convergence problems with a negative binomial distribution. Residual plots showed no heteroscedasticity and that the normal distribution fit well after data transformation. We assumed the richness data followed a Poisson distribution. Likelihood ratio tests were used to evaluate effects of removing each fixed effect. Differences were considered to be significant when *p < *0.05.

Alpha diversity and gamma diversity were calculated using the “BiodiversityR” package (Kindt, 2016) in R. Additionally, we compared each beta diversity metric across the three different land uses using one‐way Analysis of Variance (ANOVA) and Tukey contrasts for multiple comparisons of means (*p* < 0.05 = significant difference). These calculations were also done in R, and the Tukey contrasts were computed using “multcomp” package (Hothorn, Bretz, & Westfall, 2008).

In addition to measurements of alpha diversity, we computed beta diversity across the respective land uses and localities to ascertain the extent of change in community composition or species identities. There is a wide range of statistical approaches used to evaluate beta diversity, mainly focusing on species turnover, and spatial variance in community composition (Anderson et al., 2011; Jost, Chao, & Chazdon, 2011). Here, we evaluated both types of beta diversity: turnover measured the mean community dissimilarity between different sample months within the same transect, the mean community dissimilarity between different transects (summed across months) within a region and land‐use type, and the dispersion in transects within a region. As our measure of community dissimilarity, we used Horn's index, which is based on Shannon's entropy (for review see Jost et al., 2011). We square‐root‐transformed the data before evaluation to reduce the effects of particularly abundant species (Anderson et al., 2011; Anderson, Ellingsen, & McArdle, 2006). Distance indices were calculated using the *vegdist* function in the *vegan *package (Oksanen et al., 2018) in R version 3.5.0 (R Core Development Team, 2018).

To evaluate turnover, we first plotted similarity values (1 – dissimilarity) of two community samples against the difference in months between the two samples, which often follows a negative exponential decay (Anderson et al., 2011). However, we did not find evidence of any decay in similarity in our plot, except for some weak evidence of seasonality in the forest and sugarcane transects (Supporting Information Appendix S2). Therefore, to maintain the transect as the experimental unit, we took the mean difference in time for each transect. We then used this as a measure of turnover for each transect. To evaluate spatial community variance, we calculated community dissimilarity indices between the sampled communities (densities summed across all sample dates) for each transect within each land use and region. The Horn community dissimilarity indices are bounded between 0 and 1, and thus to evaluate the effects of land use and region on each, turnover and spatial beta diversity, we used a generalized linear model assuming a beta distribution. These analyses were conducted in the *betareg* package (Cribari‐Neto & Zeileis, 2010) in R (R Core Development Team, 2018). We then used Tukey's type contrasts to evaluate the difference between land‐use types using the *multcomp* package (Hothorn et al., 2008) in R (R Core Development Team, 2018).

In addition to measuring beta diversity, we investigated differences in species composition using NMDS ordination, based on a Horn dissimilarity matrix and Ward clustering. Before conducting NMDS ordination, the densities of each butterfly species were summed across the different traps and dates for a given land use, locality, and season (comprising two wet and two dry seasons), and square‐root‐transformed to reduce the impact of particularly abundant species. The (*x*, *y*) coordinates of each land use, locality, and season were then generated to identify species responsible for each cluster on the NMDS plot. These analyses were undertaken using the *vegan* package (Oksanen et al., 2018) in R, v 3.2.3 (R Core Development Team, 2018).

We calculated a habitat specificity index (*Sm*) for butterfly species collected, which is the number of individuals in the preferred habitat/total number of individuals (Brito et al., 2014). Three categories were developed as follows: (a) species that had a single habitat supporting the majority of its population: species with *Sm *> 0.9 (habitat specialist); (b) species with preference for a particular habitat but not necessarily a specialist of that habitat: species with 0.5 < *Sm* < 0.9; and (c) species that had no single habitat supporting majority of its population: species with *Sm* < 0.5 (habitat generalist). As *Sm *is sensitive to sample size (Brito et al., 2014), we used species with an individual count of five or more individuals in their population.

## 3 RESULTS

### 3.1 Patterns of abundance and richness

**Figure 1:**
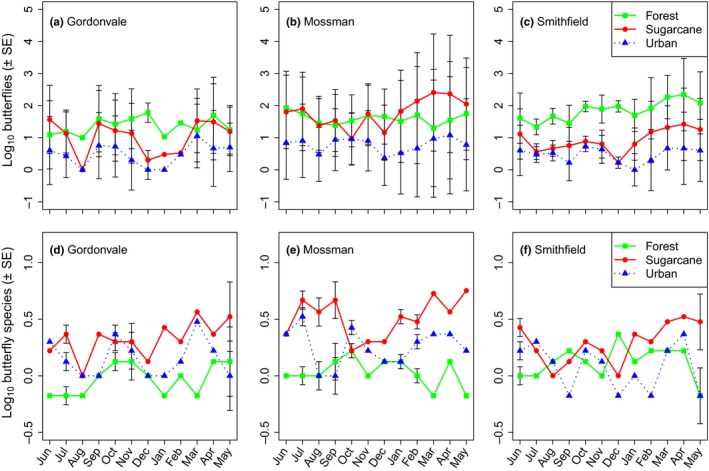
Mean (± SE) number of butterflies collected (a, c, e) and species richness (b, d, f), respectively, per land use, locality, and season. Each locality consisted of three transects within each land use, with 11 traps in each transect, and these were each sampled monthly. Number of individuals and number of species across the traps within a transect were summed on a monthly basis. Data are log_10_(*x* + 1) transformed to better illustrate patterns of abundance and richness on a consistent scale, and to match the mixed model analysis

The 12‐month survey yielded a total of 49 butterfly species and 10,460 individuals within four families across both seasons and the three localities and land uses. Each land use had a particular species dominating throughout the year. Abundances differed significantly among the three land uses (likelihood ratio test: *χ* ^2^ = 36.57, df = 2, *p < *0.0001), with the highest abundances being found in forests, and respective localities (likelihood ratio test: *χ* ^2^ = 11.63, df = 2, *p = *0.0030), with the highest abundances being found in Mossman. However, there was also a significant interaction between locality and habitat (likelihood ratio test: *χ* ^2^ = 30.56, df = 4, *p < *0.0001). The abundances of forest and sugarcane butterflies in Gordonvale interchanged throughout the survey (Figure 1a), while forest butterfly communities in Smithfield were clearly and consistently higher in number when compared to sugarcane and urban butterflies (Figure 1c). In Mossman, however, sugarcane butterflies were highest in numbers throughout most of the survey (Figure 1e).

Species richness was significantly different among land‐use types (likelihood ratio test: *χ* ^2^ = 23.89, df = 2, *p < *0.0001), with sugarcane areas supporting the most species through most of the survey period and forests supporting the least (Figure 1b,d,f). However, the magnitude of the differences depended on the locality (likelihood ratio test: *χ* ^2^ = 21.04, df = 4, *p = *0.0031), which also influenced species richness directly (likelihood ratio test: *χ* ^2^ = 8.849, df = 2, *p = *0.0120).

### 3.2 Beta diversity

**Figure 2:**
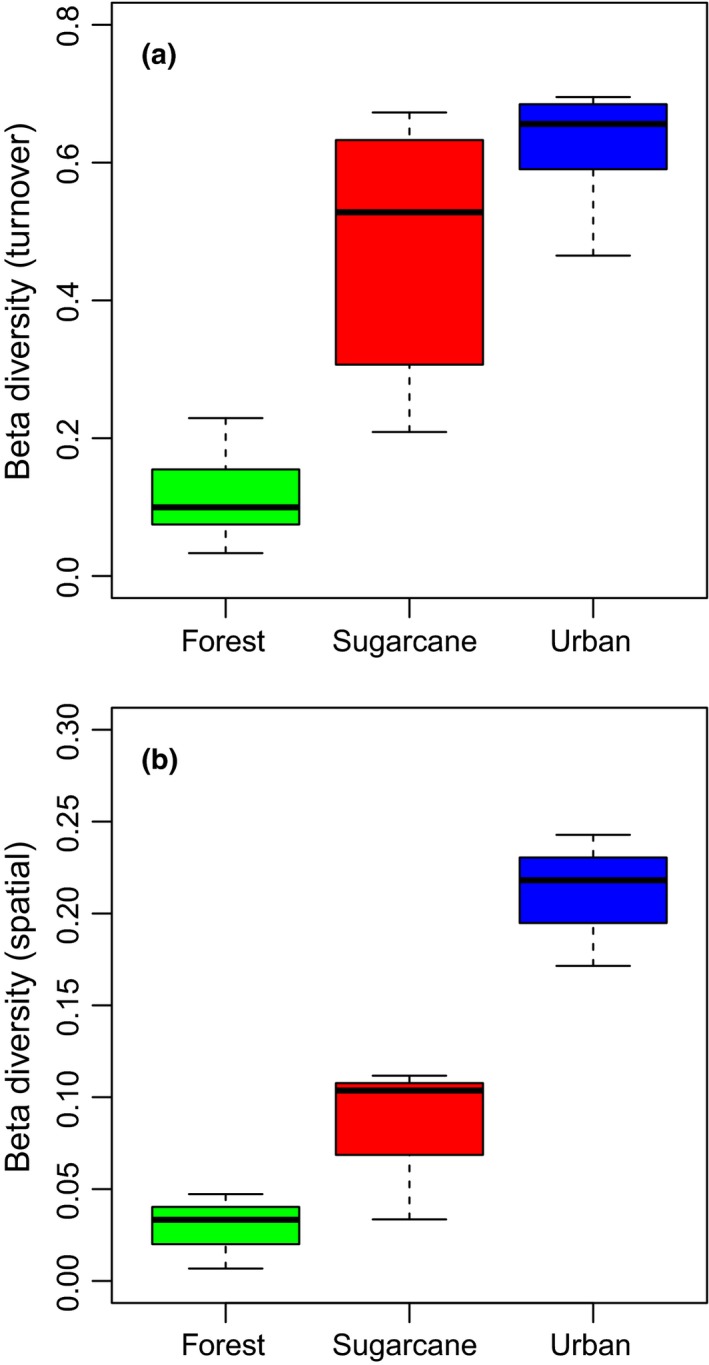
Beta diversity measured as mean Horn distance between (a) sample dates within the same transect as a measure of temporal turnover, or (b) transects within the same land use and region. There was no clear pattern in spatial turnover decay, so to evaluate turnover we present mean differences across time as a measure of change over time for a given sampled butterfly community

Beta diversity measured as turnover (the variation in species composition within a transect over time) was significantly different among the three land‐use types (beta regression Wald test: χ^2^ = 99.86, df = 2, *p* < 0.0001). The highest turnover was observed in urban environments, followed by sugarcane, and then forests (Figure 2a). Post hoc Tukey‐type tests showed that all pairwise comparisons were significant (Forest vs. Sugarcane: *p* < 0.0001; Forest vs. Urban: *p* < 0.0001; Sugarcane vs. Urban: *p* = 0.0080). There was no significant effect of region on turnover (beta regression Wald test: χ^2^ = 5.88, df* *= 2, *p* = 0.0529).

**Figure 3:**
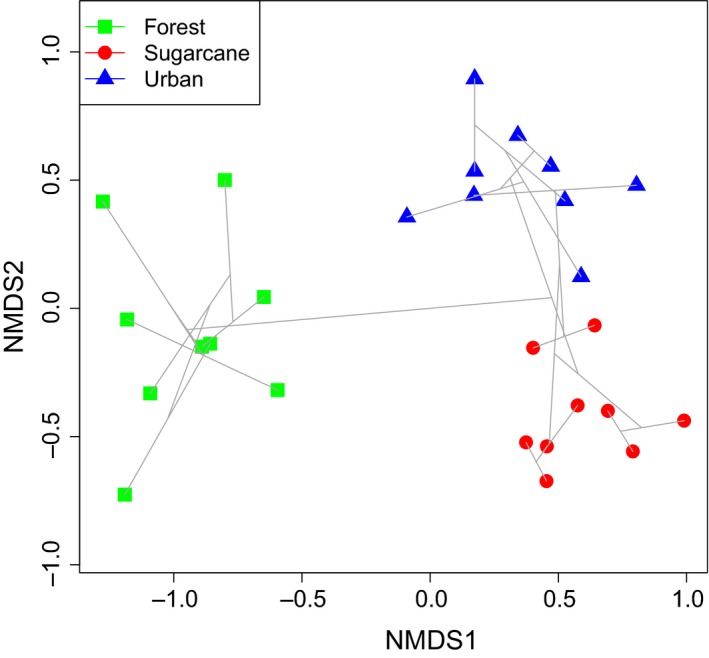
Nonmetric Multi‐Dimensional Scaling (NMDS) describing butterfly community structure (densities summed over one year of sampling), using Horn distance index. Separation in space for similar shapes represent spatial variance in a particular land‐use type (forest, sugarcane, and urban), and distances between different shapes represent differences in community structure for samples in different land‐use types

Beta diversity measured as spatial variation (the variation in species composition between transects, summed over time, within the same land‐use type and region) was significantly different among the three land‐use types (beta regression Wald test: χ^2^ = 39.30, df* *= 2, *p* < 0.0001). The highest turnover was observed in urban environments, followed by sugarcane, and then forests (Figure 3b). Post hoc Tukey‐type tests showed that all pairwise comparisons were significant (Forest vs. Sugarcane: *p* = 0.0167; Forest vs. Urban: *p* < 0.0001; Sugarcane vs. Urban: *p* = 0.0002). There was no significant effect of region on turnover (beta regression Wald test: χ^2^ = 0.62, df = 2, *p* = 0.7326).

### 3.3 Habitat specificity

Nonmetric Multi‐Dimensional Scaling suggested that forest butterfly communities differed greatly from the two human‐modified land‐use types, but that they (sugarcane and urban) also differed in their butterfly community composition (Figure 3). The habitat specificity index (*Sm*) calculations placed species into two of the three categories (habitat specialist [*Sm *> 0.9], species with habitat preference [0.5 < *Sm*>0.9], and habitat generalist [*Sm* < 0.5]), with no species found to be a generalist. There was a total of 17 specialists across the three land uses: 12 in sugarcane, four in forest, and one in urban. Additionally, a total of nine species showed habitat preference: five in sugarcane, three in urban, and one in forest (Table 1).

**Table 1:** Habitat specificity index (*Sm*) of species in the three different land uses (S = sugarcane, *F* = forest and U = urban)

Family	Species	*Sm*	Land use
Hesperiidae	Ocybadistes ardea^**^	0.93	S
	Pelopidas lyelli^**^	0.96	S
	Suniana lascivia^**^	1.00	S
	Arrhenes dschilus^*^	0.87	S
Lycaenidae	Euchrysops cnejus^**^	0.93	S
	Famegana alsulus^**^	0.92	S
	Lampides boeticus^**^	1.00	S
	Theclinesthes onycha^**^	1.00	U
	Jamides phaseli^*^	0.60	S
	Zizula hylax^*^	0.67	S
	Zizina labradus^*^	0.66	S
Nymphalidae	Acraea terpsicore^**^	1.00	S
	Junonia villida^**^	1.00	S
	Mycalesis perseus^**^	0.96	S
	Mycalesis sirius^**^	0.99	S
	Doleschallia bisaltide^**^	0.91	F
	Mycalesis terminus^**^	0.94	F
	Neptis praslini^**^	1.00	F
	Tisiphone helena^**^	1.00	F
	Hypolimnas bolina^*^	0.75	S
	Charaxes sempronius^*^	0.75	U
	Junonia hedonia^*^	0.71	U
	Phaedyma shepherdi^*^	0.60	U
	Melanitis leda*	0.56	F
Pieridae	Eurema alitha**	1.00	S
	Eurema hecabe**	0.92	S

Note

The index was calculated for each species by dividing the total number of individuals collected per land use by the total number of individuals collected in total across the three land uses. Only species that had five or more individuals in total were used in this calculation. Species are listed either as a habitat specialist (^**^) or as having a habitat preference (^*^). *Sm* values > 0.9 are classified as specialists, while those that are 0.5 < *Sm* < 0.9 have habitat preferences.

## 4 DISCUSSION

Human‐modified areas often decrease biodiversity, and increase abundance of a selected few species that are able to exploit modified habitats (Solar et al.., 2015). In contrast, our results suggest that the effect of landscape modification may depend on the type of diversity considered.  As expected, the forests in our study had highest species abundances overall when compared to human‐modified areas, but the human‐modified areas actually had higher species richness than the forests. While this finding is not consistent with those of other studies (e.g., Solar et al., 2015), it could be because of the management practices employed within the different landscapes in the Wet Tropics bioregion. Other research suggests that increasing the prevalence of weedy areas improves butterfly diversity conservation (Koh, 2008), and another study concluded that the optimal strategy for balancing butterfly conservation may include low‐intensity agriculture and preservation of field margins (Hodgson et al., 2010). Sugarcane farmers in the bioregion generally have a fallow schedule of 10%–25% of their plantation every harvesting period/year (C. Reynolds and M. Savina, personal communication). Therefore, these areas may act as havens or breeding grounds for butterflies (Pywell et al., 2004), in part due to the rapid growth of species colonizing these areas. These areas are also generally mowed regularly, potentially promoting rapid regrowth of uncultivated plants (once per month or once per 6–8 weeks – depending on the weed load; C. Reynolds and M. Savina, personal communication).

Sugarcane farmers also tend to maintain riparian vegetation along creeks and other waterways that run through or around their plantation. This vegetation could act as a corridor, as seen in other cultivation systems such as pine plantations (Haddad & Tewksbury, 2005) and ryegrass swards (Cole, Brocklehurst, Robertson, Harrison, & McCracken, 2015), allowing butterflies to move from one block to the next, or from forest to block and vice versa, thus preventing population isolation through habitat fragmentation. These high‐density populations can also act as source populations to allow for the re‐colonization of neighboring habitats thus reducing localized or even local extinction. Additionally, the waterways act as refuges for butterflies, especially during drier months when they seek out moist conditions of the drying creek beds (Braby, 2004, 2016; Cabette, Souza, Shimano, & Juen, 2017).

The urban areas in our study exhibited the highest beta diversity, as measured by both temporal species turnover and spatial variation, likely due to variation in natural green areas and residents’ landscaping preferences. Some of the cultivated plants serve as butterfly hosts flower throughout the year (e.g., *Ixora* sp.), while others have shorter flowering periods (e.g., *Callistemon* sp.). These nectar‐producing plants in the urban setting benefit from residents’ irrigation, fertilizer application and other typical gardening and landscaping activities, and may create an environment where numerous species of butterflies are able to utilize constant and multiple sources of nectar throughout the year. For example, one habitat specialist found mainly in urban areas—*Theclinesthes onycha* feeds primarily on *Cycas* sp., which was readily available due to many residents planting it as an ornamental plant in their gardens. Additionally, three species showed preference for the urban landscape (Table 1), with many of their host plants being found either as weeds or ornamental plants in urban areas (see Braby, 2004, 2016 for list of host plants). The presence of these plants provides the necessary conditions suitable for supporting several generations and in relatively high numbers when compared to the two other land management practices that were investigated.

While the sugarcane‐producing areas in our study supported the highest species richness, it produced lower beta diversity than the urban areas. Thus, while our data highlight the potential for agriculture to support high species richness, even in comparison with natural areas (Gonthier et al., 2014), agricultural landscapes have often undergone some degree of biological homogenization brought about by homogenization of resources within the physical environment (McKinney, 2006; Solar et al., 2015). As a result, the degree of community dissimilarity in human‐modified areas is often reduced when compared to forests and other natural areas (Gossner et al., 2016; Socolar, Gilroy, Kunin, & Edwards, 2016; Solar et al., 2015; Tscharntke et al., 2012). Here, we found that although sugarcane beta diversity was lower than in urban areas, it was higher than the forests. It is unclear why beta diversity was lower in forest habitats, but it may be related to the fact that we focused on variation within an eco‐region, rather than among eco‐regions. In an evaluation of bird diversity, Karp et al. (2012) found that beta diversity was higher in intensely managed agricultural areas than forests when comparisons were made within the same eco‐region, but these differences were reversed when beta diversity was estimated across biomes, due to greater variation in forest vegetation at larger scales. Thus, it is possible that the variation in forest vegetation within our study sites of the Wet Tropics eco‐region of Australia was not great enough to support high beta diversity.

Forest habitats had the highest abundance, but lower alpha and beta diversity than sugarcane and urban areas, respectively. Nonetheless, the forest habitats supported butterfly populations that differed greatly from those found in sugarcane and urban areas according to NMDS analyses. These forests are very old (Turton, 2016) and, as such, have established species adapted to the rainforest. Three species were identified as forest habitat specialists (Table 1), while one species (*Melanitis leda*) showed a preference for forest habitat despite it being the most dominant species in urban areas throughout most of the survey period. This is because numbers of *M. leda* were highest in urban areas relative to other urban species, but forests supported the highest overall abundance of this species. This species has been identified elsewhere as commonly occurring in parks and gardens, and larvae can feed on a range of grasses (e.g., Orr & Kitching, 2010). The common occurrence of *M. leda *in urban areas indicates that conditions in urban areas can reflect those found in forests, potentially through spill‐over into urban areas and the presence of forest plants in green areas. It is also interesting to note that we classified *Mycalesis terminus* as a forest specialist while two other *Mycalesis* species (*M. perseus *and *M. sirius*) are mainly found in sugarcane plantations. This evidence, which has also been found for other sister species in Guyana by Sambhu et al. (2017), is contrary to the notion that similar species behave or live in similar areas (Francesconi et al., 2013). Nonetheless, the unique compositions of forest habitats suggest that conservation of these habitats may target different species than conservation of human‐modified habitats.

Here, our study focuses primarily on fruit‐feeding butterflies, and it is worth considering how other species respond to landscape modification, since different taxonomic groups often respond differently to tropical forest disturbance (Alroy, 2017). However, butterflies have been proposed as important indicators, because they can easily be evaluated and their response can closely resemble vertebrate animals (Blair, 1999). Elsewhere, butterflies have been used to optimize land‐sharing or land‐sparing strategies to balance conservation and agricultural production (Hodgson et al., 2010). Thus, our findings may also be applied to general theory. For example, it is likely that while alpha diversity can be improved in agricultural communities, the potential for improving beta diversity may be more limited, due to low variation in vegetation composition between farms.

## 5 CONCLUSIONS

Maintaining intact natural areas remains of great importance for biodiversity conservation. However, landscapes experience different management practices which can, in turn, support different facets of biodiversity as is evident from our results. For example, urban green spaces are encouraged and maintained in many instances in our study area along with the environmentally‐friendly practices of many sugarcane farmers (such as maintaining riparian and headland vegetation that support some butterfly species and other associated species). Given that the landscape is a mosaic of different land uses, it is important to consider what aspect of biodiversity conservation needs to be achieved in particular locations. Land managers and conservation practitioners need to include all the different stakeholders that are involved in respective land uses in order to achieve landscape level conservation outcomes, thus preventing fragmentation and/or isolation that can be brought about through different land uses.

## AUTHOR CONTRIBUTIONS

HS conceived the main idea and designed the study, collected and analyzed the data, created the figures and tables, and wrote the manuscript. AN assisted in collecting and analyzing data, creating figures and tables, and editing drafts of the manuscript. ST reviewed and edited drafts of the manuscript. TN assisted in the logistics of data collection, assisted in data analyses, and reviewed and edited drafts of the manuscript.

## DATA ACCESSIBILITY

Butterfly abundance data are available on Dryad (DOI accession number: https://doi.org/10.5061/dryad.6gd8hb5).
