# Supplementary figures and images for: Trade‐offs for butterfly alpha and beta diversity in human‐modified landscapes and tropical rainforests
Source: Ecol Evol. 2018 Dec 6;8(24):12918–28. doi: 10.1002/ece3.4732 (PMC6309007; doi:10.1002/ece3.4732)

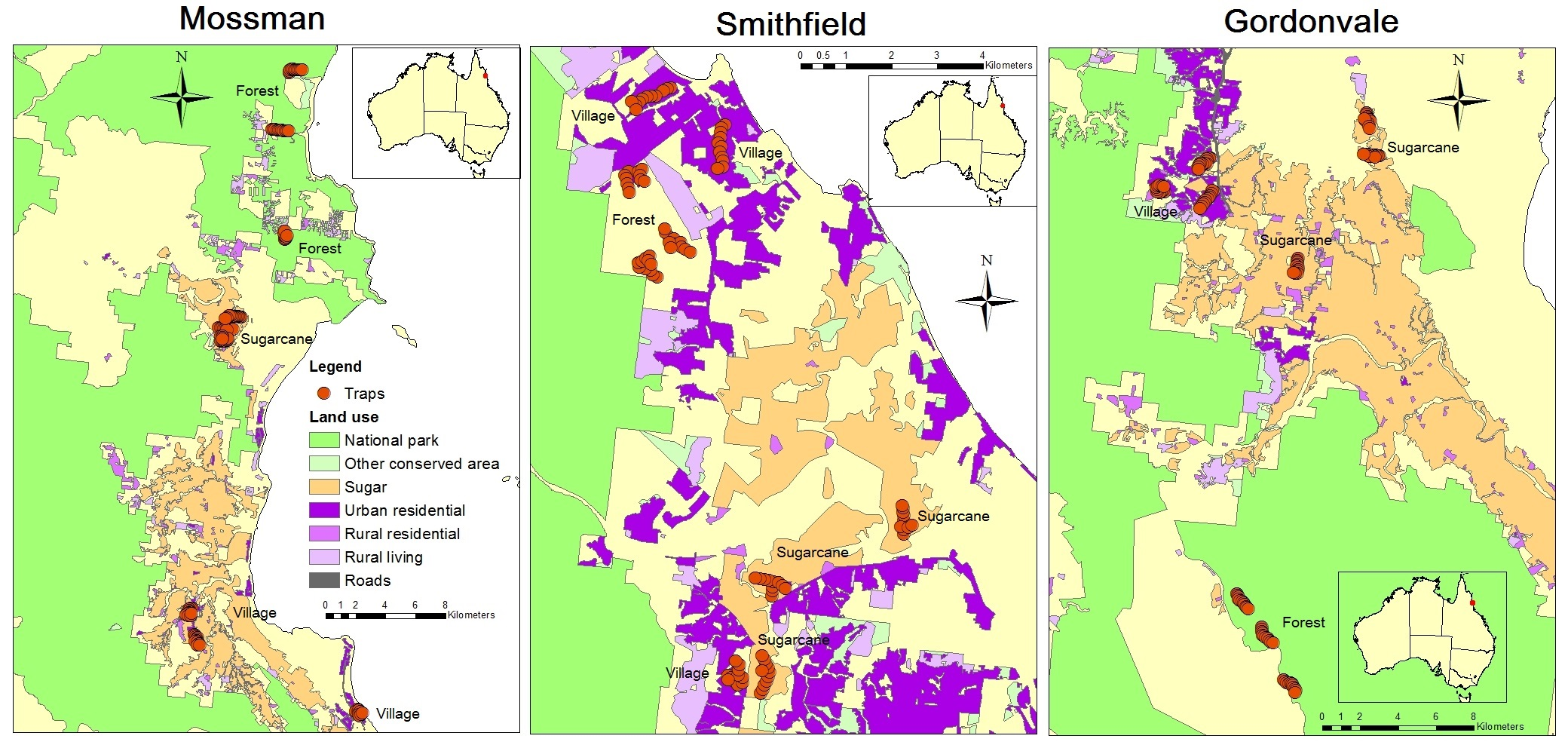

Supplement: Supplementary file 1 [file ECE3-8-12918-s001.tif]

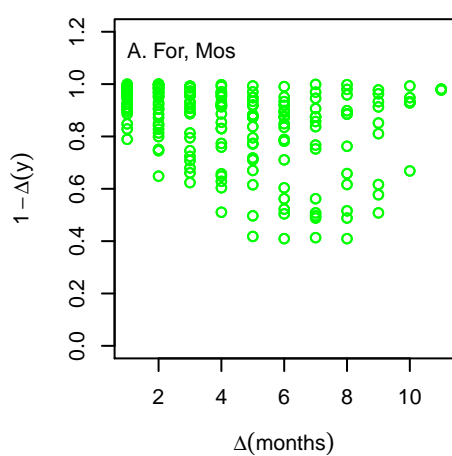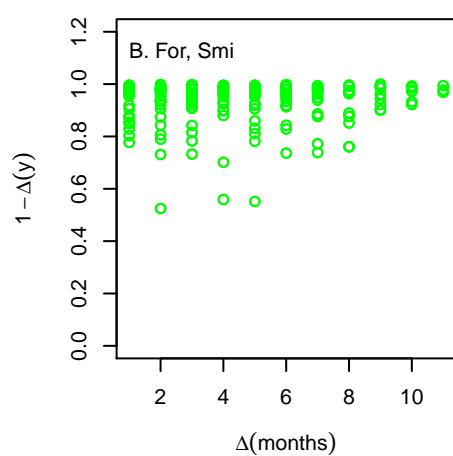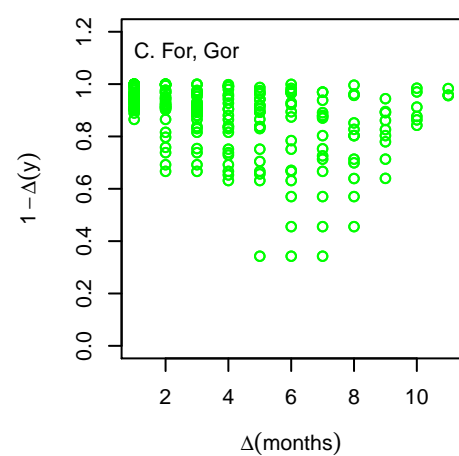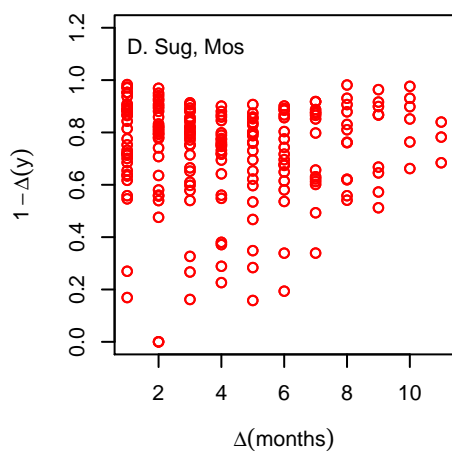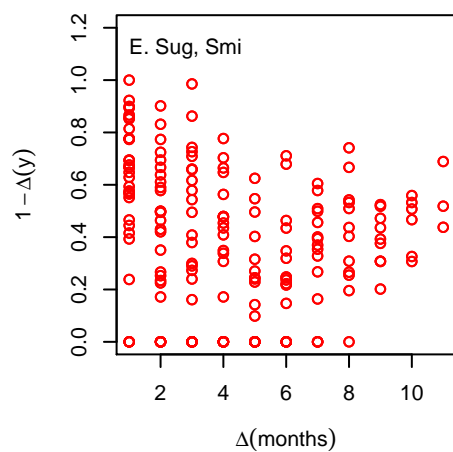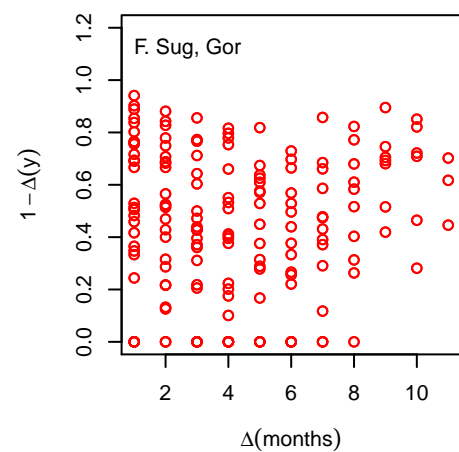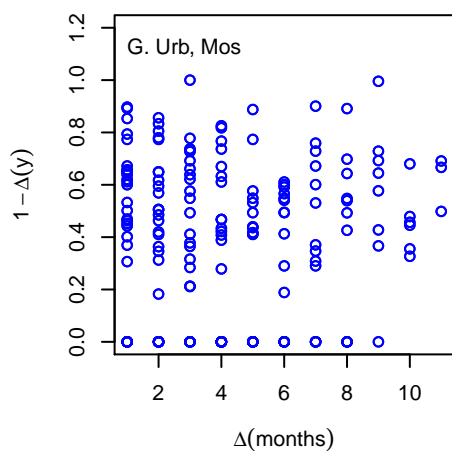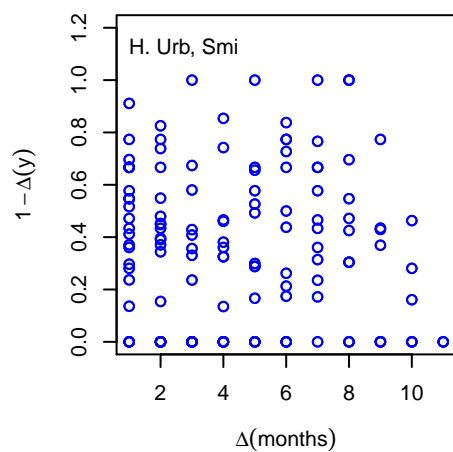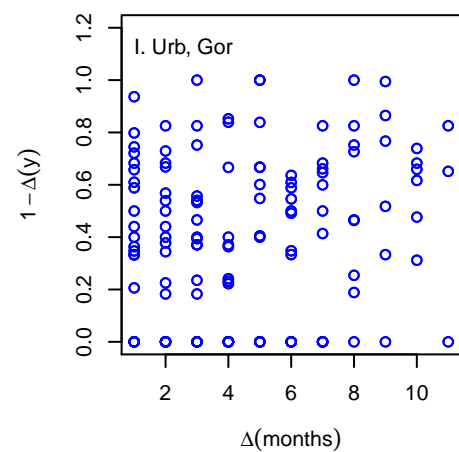

Supplement: Supplementary file 2 [file ECE3-8-12918-s002.pdf]
